# The impact of self-stigma on quality of life in individuals with severe mental illness

**DOI:** 10.1192/j.eurpsy.2026.11708

**Published:** 2026-07-31

**Authors:** M. Touzri, Y. Rebei, S. Ben Othmene, H. Mami, A. Aissa

**Affiliations:** Faculty of medicine of Tunis, Nabeul regional hospital, Nabeul, Tunisia

## Abstract

**Introduction:**

Self-stigma is defined as the process by which individuals with mental illness internalize negative societal stereotypes, expect rejection from others, and feel alienated from society. This internalized stigma can have detrimental effects on self-esteem, social relationships, treatment adherence, and overall quality of life.

**Objectives:**

The aim of this study was to assess the impact of self-stigma on the quality of life among individuals living with severe mental illness.

**Methods:**

We conducted a cross-sectional descriptive study involving 70 individuals with severe mental illness. The *Internalized Stigma of Mental Illness* (ISMI) scale was used to assess self-stigma, and the *World Health Organization Quality of Life – BREF* questionnaire was used to evaluate quality of life. Data were analyzed using SPSS version 26, applying Pearson’s correlation and multiple linear regression analyses.

**Results:**

Seventy patients were included. The mean age of participants was 46.2 ± 13 years with a gender ratio of 1,3. Moderate to severe stigma was found in 57% of patients, with a mean score of 2.6 ± 0.5.

The mean WHOQOL-BREF scores in our sample were 50.5 for the physical health domain, 45.7 for the psychological domain, 37.6 for social relationships, and 37.5 for the environment domain.

All domains of quality of life were inversely correlated with the mean of self-stigma score. No significant correlation was found between self-stigma and the global health item of the WHOQOL-BREF.

**Conclusions:**

Self-stigmatization was markedly present among patients with severe mental disorders and negatively impacted quality of life across all domains. These findings highlight the importance of interventions aimed at raising awareness among patients and society, and combating mental illness stigma.

**Disclosure of Interest:**

None Declared

